# Commencement

**Published:** 1879-05

**Authors:** 


### COMMENCEMENT.

The fourth annual commencement of the Dental College of the University of Michigan was held in the hall of the University, on Wednesday, March 26th, 1879. This was held at the same time, and in connection with the commencement exercises of the law and medical departments.

Interesting addresses were delivered before the graduates of the dental class, on the evening previous to the commencement, by Dr. Thomas, of Detroit, and Prof. Watling.

At the commencement, an address, to professional men, was delivered by ex-Gov. Blair, which was replete with good thoughts and happy suggestions.

The degree of D. D. S. was conferred by the president of the University, Dr. J. B. Angell, upon:

David M. Cattell, Cadiz, O.; William Henry Dorrance, Jackson; Frank Oscar Gilbert, Bay City; Clark Lowell Gregory, Albion; Herbert Frank Harvey, Cleveland, O.; George Thomas Higgins, Brooklyn, N. Y.; James Henry Kennicott, Arlington Heights, Ill.; Edwin Jacob Lilly, Circleville, O.; Henry Bristor Orr, Mansfield, O.; Howard Treat Sackett, Tallmadge, O.; Samuel Bartlett Short, La Grange, Ind.; E. Frank Sites, Fort Wayne, Ind.; Frank H. Waldron, Brimfield, O.; Corydon L. Ford Wall, Cambridge O.; Frank B. Wells, Ovid; total fifteen.

